# *In Situ* EC-AFM Study of the Initial Stages of Cathodic Corrosion of Pt(111) and Polycrystalline Pt in Acid Solution

**DOI:** 10.1021/acs.jpclett.3c00579

**Published:** 2023-05-24

**Authors:** Xiaoting Chen, Marc T. M. Koper

**Affiliations:** ‡ School of Materials Science and Engineering, Beijing Institute of Technology, Beijing 100081, P. R. China; † Leiden Institute of Chemistry, Leiden University, PO Box 9502, 2300 RA Leiden, The Netherlands

## Abstract

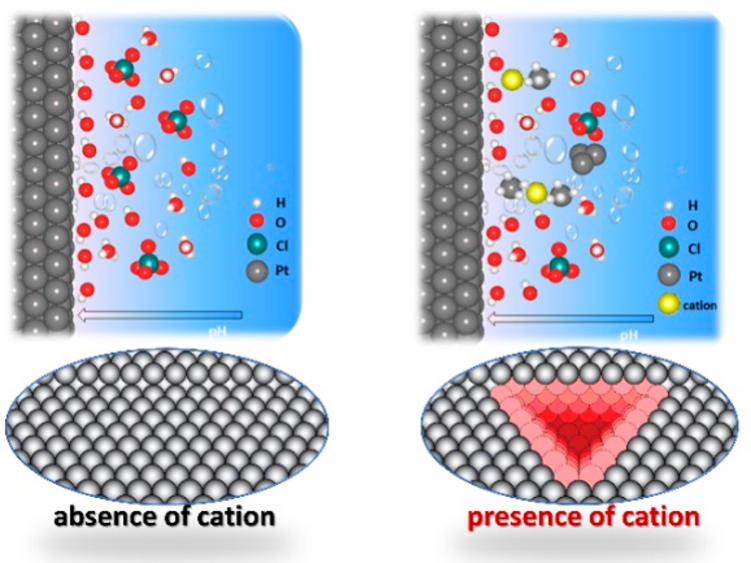

An atomic scale understanding of the surface degradation mechanism during cathodic corrosion of a platinum electrode is still lacking. Here, we present results of surface structural changes observed during cathodic polarization of a polycrystalline Pt electrode and single crystalline Pt(111) in acid electrolytes in the absence and presence of cations (Na^+^) by *in situ* electrochemical atomic force microscopy (EC-AFM) imaging. The electrolyte cation is proved to be a prerequisite to trigger cathodic etching of the polycrystalline Pt surface. Further examination of the evolution of electrochemical signals and distinct surface structural transformations of an atomically defined Pt(111) single-crystal electrode during cathodic corrosion reveals clearly that the roughening process commences at the under-coordinated sites of the Pt(111) surface. The created triangular-shape pattern, actually a 100-oriented pit in a 111-terrace, grows primarily laterally in the initial regime, while prolonged cathodic corrosion leads to the existing etching pits growing in depth until ultimately they coalesce with each other, generating a highly roughened surface.

Platinum is massively employed as a catalyst in a great number of electrochemical devices such as fuel cells and electrolyzers, where it catalyzes the hydrogen evolution/oxidation reaction (HER/HOR) and the oxygen reduction reaction (ORR).^1−5^ However, the widespread application of these devices is hampered by reactivity loss and suboptimal durability of the electrocatalysts, caused by structural destruction and/or dissolution of platinum during long-term employment. Electrochemical processes of anodic and cathodic corrosion etch platinum electrodes when very positive and negative potentials are applied, respectively. The oxidation of polycrystalline platinum commences by the formation of a surface layer of chemisorbed hydroxide and/or oxide. The subsequent reduction of the Pt oxide leads to a severe surface restructuring/roughening and nanoparticle formation, which has been ascribed to a dissolution–reprecipitation process involving anodic corrosion. The elucidation of the surface morphology evolution (reconstruction and/or nanoparticle redeposition) of Pt electrodes^6−10^ and the possible Pt nanoparticle dissolution^11−13^ after/during anodic corrosion has been amply documented. Recent work showed the evolution of the overall roughness of a Pt(111) single-crystal electrode and its correlation to the total electrochemical signal, as studied by *in situ* electrochemical scanning tunnelling microscopy (EC-STM).^14^

Previous research on cathodic corrosion of platinum electrodes has been reviewed recently.^15^ Cathodic corrosion is not only a degradation pathway that would decrease the stability and long-term performance of Pt-based catalysts, but it also appears as a promising synthetic route for the efficient and facile preparation of Pt nanoparticles with preferred size and facets, which could be utilized as high-performance catalysts in, for example, methanol oxidation and nitrite reduction.^16−21^ The morphological changes (etching patterns) of the single-crystalline platinum electrode surface after cathodic treatment in aqueous solution present improved catalytic activities for, e.g., oxygen reduction and glycerol oxidation reaction, compared to the untreated surface.^22^ To explore protocols realizing protection against cathodic corrosion of Pt and/or to develop Pt-based catalysts that are substantially more active after cathodic corrosion, we must focus on a fundamental understanding of this phenomenon. It has been found previously that cathodic corrosion of Pt is anisotropic, i.e., facet sensitive, and that irreducible cations, both metallic cations and organic cations (ammonium and tetraalkylammonium), play a decisive role in cathodic corrosion,^18,23,24^ in contrast to anodic corrosion. More specifically, the presence of cations like Na^+^ and NH_4_^+^ is assumed to be a prerequisite for cathodic corrosion of platinum,^18^ and the surface patterning process as well as the creation of Pt nanoparticles dissolved into solution show a strong dependence on cation identity and concentration.^19,24^

Work performed on platinum spherical single-crystal electrodes has confirmed that cathodic corrosion of Pt is highly anisotropic and the (111) facet is much more sensitive to cathodic corrosion than other basal planes^25^ (whereas the (111) facet is the most robust surface during anodic corrosion^26^). So far, most studies investigated cathodic corrosion of Pt by characterizing the surface morphologies (or the nanoparticles it generated in solution) *ex situ*, primarily by scanning electron microscopy (SEM) before and after cathodic corrosion. However, the obtained SEM images for Pt surfaces characterized after cathodic corrosion are, usually, on the micrometer scale resolution and additionally provide no insight into the three-dimensional nature (depth of pits) of etching patterns. An additional complication of monitoring etch structures *in situ* during cathodic corrosion is the vigorous gas-evolving process of hydrogen evolution which accompanies cathodic corrosion. To unravel the intricacies of the roughness evolution of platinum, *in situ* monitoring of the etch structures on atomic well-defined surfaces during cathodic corrosion is desired. Electrochemical atomic force spectroscopy (EC-AFM) is a powerful tool for the real-space characterization of catalysts under realistic electrochemical reaction conditions. The utilization of *in situ* EC-AFM showed polycrystalline Pt surface evolution during anodic corrosion^7,8^ and more recently provided a direct visualization of the surface structure evolution of a Cu(100) single-crystal electrode during CO_2_ reduction.^27,28^

In this work, we performed cathodic corrosion of polycrystalline Pt in acid solution without and with the addition of a metal cation like Na^+^ and explore the pivotal role played by the involved cation (Na^+^). In the absence of irreducible cations, cathodic corrosion does not take place. We also monitored the evolution of the surface structural changes of an atomically well-defined Pt(111) single-crystal electrode during cathodic corrosion in perchloric acid containing sodium cations by *in situ* EC-AFM. We elucidate how the morphological evolution relates unequivocally to the underlying atomic scale structure of the Pt(111) single-crystal electrode during cathodic corrosion. The new insights in the degradation process of Pt caused by cathodic polarization are of importance for the preparation of improved Pt electrocatalysts with preferred sites and facets by cathodic corrosion and to the rational design of operating electrolytes with an expanded lifespan of Pt electrodes.

*Cathodic Corrosion of Polycrystalline Pt Electrode in Acid Solution*. We first describe the cathodic corrosion of a polycrystalline Pt electrode in acid solution in the absence and presence of Na^+^ cations. For a platinum electrode, the use of cyclic voltammetry, especially the so-called “hydrogen region” in sulfuric acid, is a well-established “fingerprint” for characterizing the electrode surface structure.^29^Figure 1a shows the cyclic voltammogram of polycrystalline Pt electrode in 0.1 M H_2_SO_4_ between 0.05 and 0.65 V_RHE_ at a scan rate of 50 mV/s. It shows the characteristic features of the blank voltammogram of polycrystalline Pt electrode in 0.1 M H_2_SO_4_ (black curve): a broad H adsorption–desorption feature corresponding to the 111-terrace (0.05 < *E* < 0.30 V_RHE_); step-related voltammetric peaks involving the replacement of H by OH on 110-step sites (*E* = 0.13 V_RHE_) and 100-step sites (*E* = 0.27 V_RHE_),^30,31^ respectively; and a broad feature between 0.30 and 0.40 V_RHE_ corresponding to H adsorption–desorption on 100-terrace sites.^29^ The voltammogram indicates a higher fraction of 100-sites as a result of numerous cathodic corrosion studies to this electrode, and the inductive heating preparation might therefore not have recovered to a typical standard polycrystalline Pt electrode profile (Experimental Methods). Figure 1b shows the surface morphology of the polycrystalline Pt working electrode imaged by *in situ* EC-AFM at a potential of ca. 0.50 V_RHE_ in the Pt double-layer region. The surface is not atomically flat and presents terraces with sawtooth-like steps uniformly covering the whole imaging frame, which is assigned to a faceting induced by flame annealing.^7,8^Figure S2 also displays the pristine polycrystalline Pt electrode at a larger image frame of 5 × 5 μm. The AFM images appear to resemble images of a stepped Pt(100) electrode,^32^ but they can only indicate terraces with sawtooth-type successive steps while the identification of specific orientations/facets for terraces and steps are outside of the resolution of the EC-AFM images. Next, we performed a strongly cathodic treatment of the polycrystalline Pt working electrode in 5 M HClO_4_ at −4.0 V_RHE_ for 5 min. Figure 1a shows the cyclic voltammogram of the polycrystalline Pt working electrode after (red curve) cathodic corrosion in 5 M HClO_4_. Compared with the original voltammogram of Pt (black curve), subtle changes of surface structure are indicated. The AFM image in Figure 1c shows that the surface morphology of polycrystalline Pt electrode obtained after cathodic polarization is equally subtly changed compared to the image in Figure 1b, with a few granular bright dots and relatively small changes near the sawtooth-like steps. The area with scar-like defects which appear in the AFM images of the original Pt electrode are not in the exact same position due to thermal drift (as shown in Figure S2). We have argued recently, based on experiments and density functional theory calculations, that a high coverage of hydrogen present on the Pt surface at low electrode potentials (i.e., below 0.17 V_RHE_) promotes the reconstruction of 110-step sites to under-coordinated “corner” sites and/or 100-step sites.^33^ The changes near sawtooth-like step sites observed in Figure 1c could perhaps be partially ascribed to this step faceting. Owing to other factors, such as bubble formation and heat generation, the deposition of trace impurities or contaminations may account for the granular bright dots in Figure 1c. Most importantly, however, we can conclude that strong cathodic polarization of the polycrystalline Pt surface in 5 M HClO_4_ does not lead to extensive large-scale surface changes.

**Figure 1:**
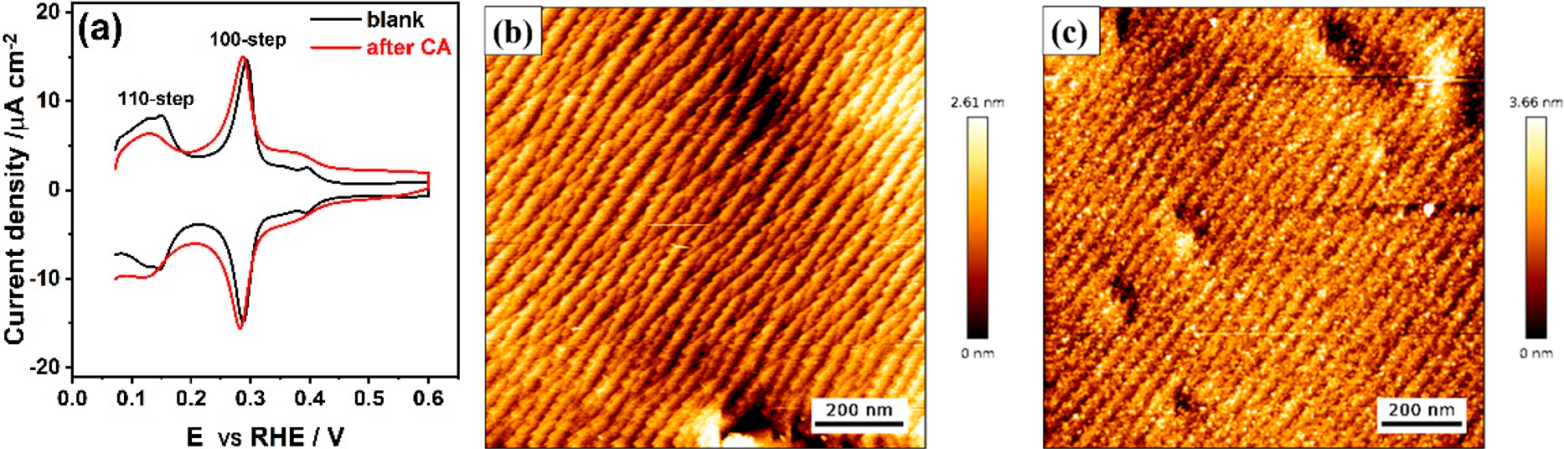
*In situ* EC-AFM results of a polycrystalline Pt electrode before and after cathodic corrosion in pure acid electrolyte. (a) Cyclic voltammograms of polycrystalline Pt electrode recorded in 0.1 M H_2_SO_4_ before (black curve) and after (red curve) cathodic corrosion at −4.0 V_RHE_ in 5 M HClO_4_ for 5 min. Scan rate: 50 mV/s. Corresponding AFM height images of the polycrystalline Pt electrode surface imaged (b) before and (c) after cathodic polarization.

To adequately elucidate the role of the cation, we carried out cathodic polarization of a polycrystalline Pt electrode in 5 M NaClO_4_ + 0.1 M HClO_4_ at −4.0 V_RHE_ for 5 min. As can be seen in Figure 2a, the cathodic treatment of the Pt polycrystalline electrode in acid solution in the presence of Na^+^ cations causes substantial changes in the blank voltammogram. First, the peak related to 100-step sites (*E* = 0.27 V_RHE_) has increased while the peak associated with 110-step sites (*E* = 0.13 V_RHE_) has almost completely disappeared. Second, a higher current is observed between 0.30 and 0.40 V_RHE_ indicating an increase in the number of 100-terrace sites. Figure 2b shows the pristine surface morphology of a polycrystalline Pt electrode, in good agreement with Figure 1b and the literature,^7,8^ which confirms the cleanliness and efficacy of the preparation of our working electrode and AFM setup. The AFM image in Figure 2c clearly shows that the surface of the polycrystalline Pt electrode undergoes an extensive roughening after cathodic treatment in acid electrolyte containing 5 M Na^+^: the formation of etching pits with ca. 5 nm depth, and some of the pits display a triangular shape similar to that shown in previous reports of cathodic corrosion of a Pt wire in 10 M NaOH.^23,24^ In contrast to the roughening phenomena of Pt during anodic corrosion,^7,8,14,34^ cathodic corrosion leads to more 100-type features instead of 110-type features and etching pit formation rather than Pt nanoparticle deposition. Electrolyte analysis after cathodic treatment of Pt electrodes has been studied extensively in earlier studies using various characterization methods, confirming that a considerable amount of Pt dissolves into solution as nanoparticles.^15,17,19−21,24^ The significant observation here is that the etching patterns, i.e., pits and holes, caused by Pt dissolution from the polycrystalline Pt surface during cathodic treatment, only occur in perchloric acid containing sodium cations. We also note that while the bulk pH is acidic (and different between the two experiments in Figures 1 and 2), the near-electrode pH during cathodic corrosion is extremely alkaline due to the high hydrogen evolution current. Therefore, we cannot relate the differences in Figures 1 and 2 to different bulk pH or to different buffering abilities.

**Figure 2:**
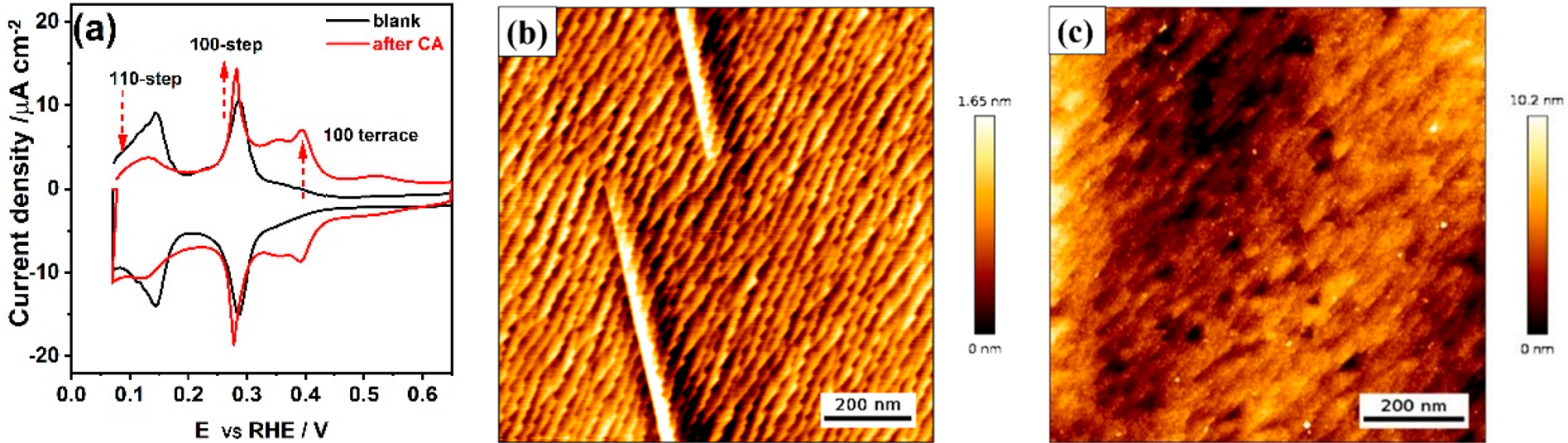
*In situ* EC-AFM results of a polycrystalline Pt electrode during cathodic corrosion in acid electrolyte containing Na^+^ cations. (a) Cyclic voltammograms of polycrystalline Pt electrode recorded in 0.1 M H_2_SO_4_ before (black curve) and after (red curve) cathodic corrosion at −4.0 V_RHE_ in 0.1 M HClO_4_ + 5 M NaClO_4_ for 5 min. Scan rate: 50 mV/s. Corresponding AFM height images of the polycrystalline Pt electrode surface obtained (b) before and (c) after cathodic polarization.

The key intermediates of cathodic corrosion have so far remained elusive.^20^ Recent computational investigations^24,35,36^ postulate that cation-stabilized negatively charged platinum-hydride PtH_*x*_^*y*–^ species act as the intermediate of cathodic corrosion of a Pt electrode. The experiment illustrated in Figure 2 clearly shows the importance of the alkali (irreducible) cation in the cathodic corrosion process. We note that previous experimental work typically performed cathodic corrosion studies in (strongly) alkaline media. In such electrolytes, (alkali) cations are automatically present. During cathodic corrosion in acidic media, as performed above, the local pH near the electrode will become (very) alkaline, but in the absence of irreducible cations in the bulk solution, this local alkalinity is not accompanied by a high cation concentration. Our experiment here illustrates that the key factor in initiating cathodic corrosion is not the (local) alkalinity of the solution, but the combination of a strong negative potential with the presence of irreducible cations.

*Initial Stages of Cathodic Corrosion of Pt(111) Single-Crystal Electrode*. Given that cathodic corrosion of a Pt electrode is highly anisotropic and that the Pt(111) is the facet most sensitive to cathodic corrosion,^25^ we focus on the Pt(111) surface as a model system to investigate the atomic-level details of the surface degradation under cathodic polarization. *In situ* EC-AFM is employed to capture the evolution of electrochemical signals and morphological changes of the Pt(111) surface during cathodic corrosion in 5 M NaClO_4_ + 0.1 M HClO_4_ for different periods. Figure 3a shows the blank voltammogram (black curve) of Pt(111) in 0.1 M H_2_SO_4_: a broad H ad/desorption feature on the 111-terrace (0.05 < *E* < 0.35 V_RHE_), the (bi)sulfate ad/desorption between 0.35 and 0.60 V_RHE_, and a single sharp peak observed at 0.50 V_RHE_ which arises from the order–disorder transition of the (bi)sulfate adlayer^37^ and which only occurs on wide and well-prepared 111 terraces with very low step density. The total charge of ca. 160 μC cm^–2^ arises from a 2/3 monolayer of hydrogen desorption on the Pt(111) electrode (1 ML of one monovalent adsorbate adsorbed per surface atom, or 1.5 × 10^15^ atoms cm^–2^, is exactly 240 μC cm^–2^), which is obtained by integrating the anodic, double-layer-corrected current between 0.05 and 0.35 V_RHE_ (as indicated in Figure 3a).

**Figure 3:**
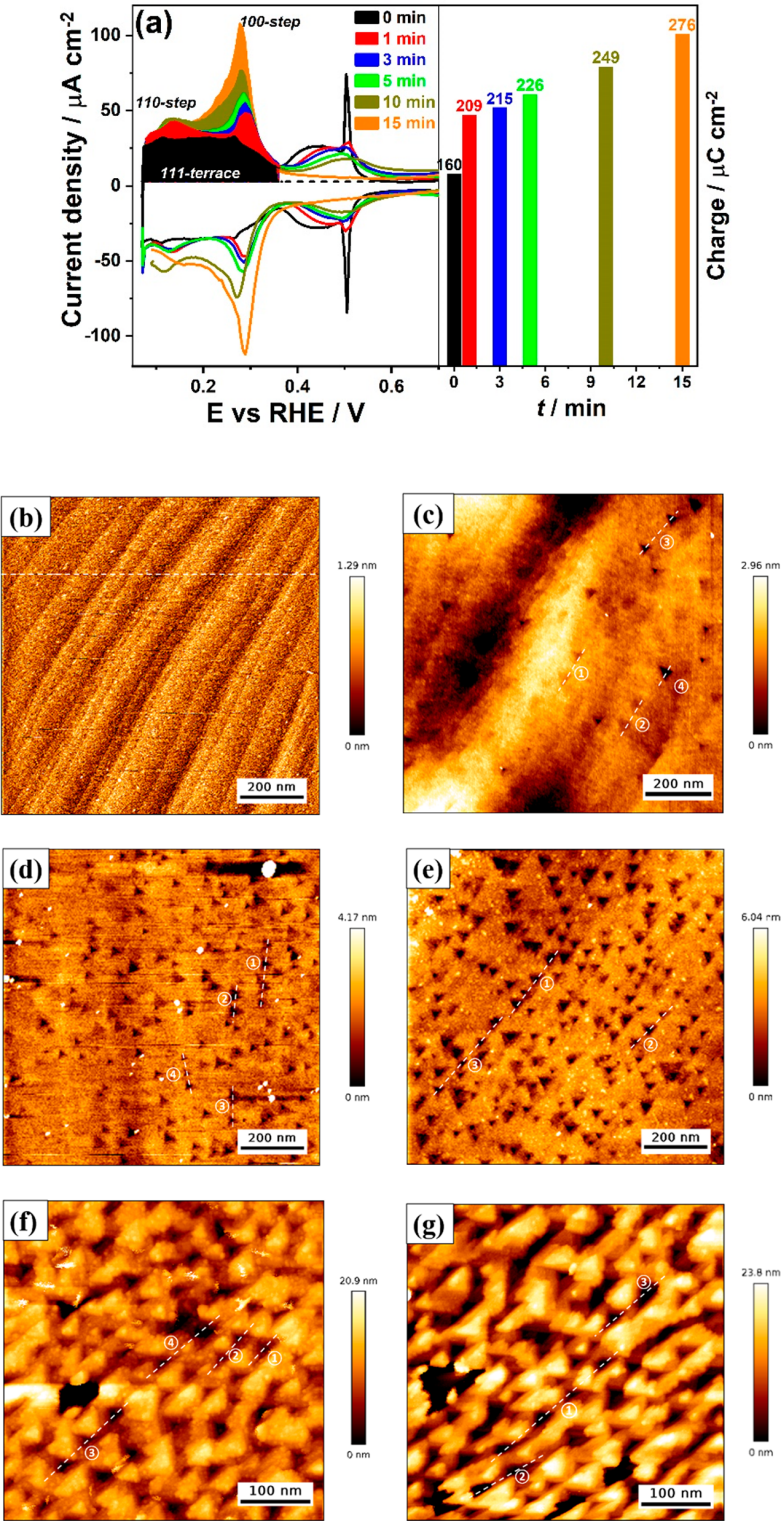
*In situ* EC-AFM results of a Pt(111) single-crystal electrode during cathodic corrosion in acid electrolyte containing Na^+^ cation. (a) Cyclic voltammograms of Pt(111) single-crystal electrode recorded in 0.1 M H_2_SO_4_ before (black curve) and after cathodic polarization at −4.0 V_RHE_ in 5 M NaClO_4_ + 0.1 M HClO_4_ for 1 min (red curve), 3 min (blue curve), 5 min (green curve), 10 min (olive curve), and 15 min (orange curve). Scan rate: 50 mV/s. The bar diagram on the right side shows the integrated overall H desorption charge as a function of the time of cathodic treatment. AFM height images of the Pt(111) electrode surface (b) before and after cathodic polarization for (c) 1 min, (d) 3 min, (e) 5 min, (f) 10 min, and (g) 15 min. Dashed lines indicate the sections along which height lines have been taken, shown in Figure 4.

Figure 3b shows the pristine surface of Pt(111) imaged by *in situ* EC-AFM with holding the potential in the double-layer region at 0.50 V_RHE_. The surface is composed of atomically flat terraces some hundreds of Pt atoms wide (width of 20–100 nm), separated by steps/defects. It is similar to the state-of-the-art Pt(111) surfaces reported by *in situ* EC-STM^14,38^ and EC-AFM,^39^ respectively, in acid electrolytes. Additionally, Figure S3 displays the pristine Pt(111) surface with 111-terraces divided by steps/defects at a larger image frame of 5 × 5 μm. To further quantify the morphology of the initial stages of the Pt(111) electrode during cathodic corrosion, we extracted the lateral and vertical (depth) size of the etching patterns from the AFM images. The height lines in Figure 4 were taken along identical terraces in the image frame, as indicated by dashed lines in Figure 3. The height line of the original surface (Figure 4, black line) shows a well-prepared flat 111-terrace with height variations of ca. 0.5 nm.

**Figure 4:**
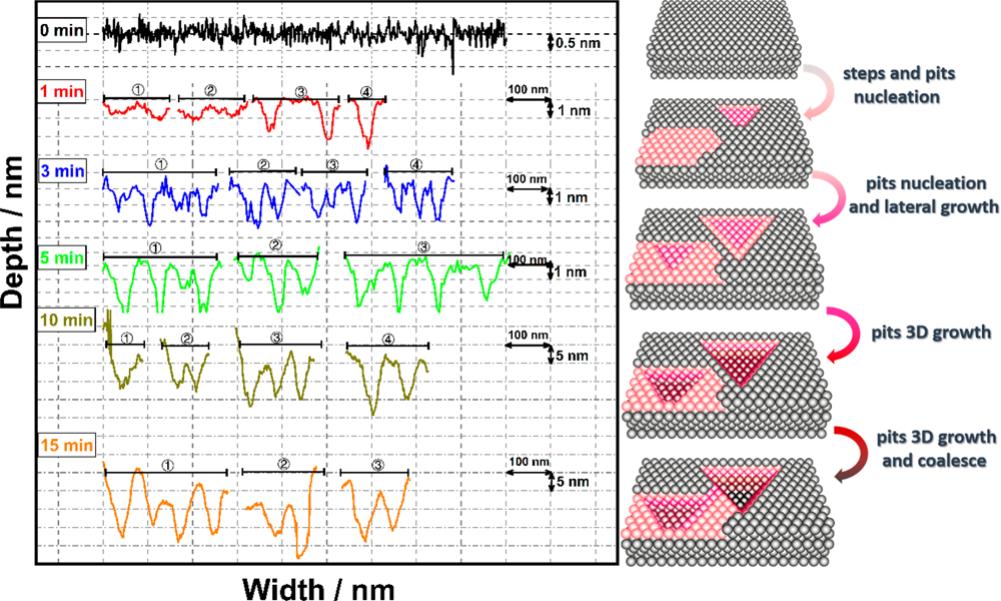
Lateral and EC-AFM estimated depth size of the created etching patterns as a function of cathodic polarization time. Height lines were extracted from the AFM images obtained during cathodic corrosion of Pt(111) electrode for various periods. The numbering correspond to the lines indicated in Figure 3. The surface models on the right depict very schematically the surface structural transformation process of the Pt(111) single-crystal electrode during cathodic corrosion. The etch pits are not drawn to scale; the models are only meant to give a rough idea of the various stages in the development of the pits.

Figure 3a shows the presence of 110- (*E* = 0.13 V_RHE_) and 100-step (*E* = 0.27 V_RHE_) sites after cathodic corrosion at −4.0 V_RHE_ in 5 M NaClO_4_ + 0.1 M HClO_4_ for 1 min (red curve), with the overall charge of H desorption region increasing from 160 to 209 μC cm^–2^. The sulfate phase transition peak at 0.50 V_RHE_ has disappeared (Figure 3a, red curve) due to the destruction of the wide 111-terraces during cathodic corrosion. The corresponding surface morphology imaged by *in situ* EC-AFM in Figure 3c reveals the presence of a few triangular-shaped etching patterns after performing cathodic polarization of the Pt(111) electrode for 1 min. The triangular pit shape could be associated with the formation of (100) or (111) symmetry walls; the increase in the (100) type sites in the CV suggests that the walls have (100) symmetry (Figure 3a). As mentioned above, we expect these etch patterns to be associated with the formation of surface hydride phases, which are unstable (dissolve) in the presence of irreducible cations (such as sodium) in the electrolyte.

Figure 3a shows the gradual increase of the density of 100-step sites with prolonged cathodic corrosion period to 3 min (blue curve) and 5 min (green curve), respectively, while the density of 110-step sites remains the same as that after 1 min of cathodic corrosion (red curve).

After the cathodic treatment of the Pt(111) electrode for 1 min, the 111-terrace is covered with crystallographic etching patterns: initial etching pits show a lateral size of 45 ± 5 nm and EC-AFM measured depth of 0.2–1 nm (we refer to the depth as “EC-AFM measured depth”, as accurate depth measurements with AFM are challenging), suggesting primarily lateral etching at the initial stages of cathodic corrosion. Upon prolonged cathodic polarization to 3 and 5 min, the density of well-defined triangular etching pits increases, but they still show a lateral size of 40–50 nm but now with an EC-AFM measured depth of 2–2.5 ± 0.5 nm (Figure 4, blue and green line). Figure 3a shows the overall charge of the H desorption region increases to 215 and 226 μC cm^–2^ after cathodic corrosion for 3 and 5 min, respectively, and the (bi)sulfate ad/desorption between 0.35 and 0.60 V_RHE_ has further diminished due to the destruction of 111-terrace sites during cathodic corrosion. Notably, Figure 3c–e shows how the etching pits appear distributed in straight lines, indicating that the pits may nucleate from step sites, although we have no atomically resolved evidence for this. Upon increasing cathodic corrosion to 10 min, Figure 3f and the corresponding height line in Figure 4 (olive line) show how the surface has almost completely filled with etch pits with an EC-AFM measured depth of 10 ± 5 nm. The disappearance of the reversible (bi)sulfate ad/desorption process between 0.35 and 0.60 V_RHE_ after cathodic polarization of 15 min (Figure 3a, orange curve) signifies that the original 111-terrace sites have been totally destroyed. In the corresponding AFM image (Figure 3g), the original 111-terrace can indeed no longer be recognized. The etching patterns now appear to grow mainly vertically into the surface (pit depth of 15 ± 5 nm as shown in Figure 4, orange line); the pits uniformly cover the surface and have commenced to coalesce with each other. Cathodic corrosion of a Pt electrode is highly anisotropic and strongly cation-dependent.^26^ Further fundamental understanding requires *in situ* monitoring of the etch structures during cathodic polarization on Pt single-crystal electrodes with different step identity and density, in electrolytes with different types of cations.

Here, we have presented *in situ* EC-AFM characterization results of the cathodic corrosion of a polycrystalline Pt electrode and a Pt(111) single-crystal electrode, respectively, during cathodic polarization at −4.0 V_RHE_ in acidic electrolyte. Our study shows the importance of irreducible cations in the electrolyte in triggering cathodic corrosion. In their absence, no large-scale cathodic corrosion takes place. Experiments with the Pt(111) single crystal illustrate in detail how cathodic corrosion gradually modifies the surface. Triangular shaped cathodic corrosion pits nucleate at step sites, as evidenced by the fact that they are lined up and all have the same orientation. The shape of the pits corresponds to the generation of (100)-type sites, in agreement with the voltammetry fingerprint. Initially, these etch pits grow mainly horizontally, with a lateral size of ca. 50 nm and a depth of ca. 1–2 nm. Once the etch pits “touch”, they continue to grow mainly vertically. This eventually leads to a highly roughened surface in which the original Pt(111) structure is no longer recognizable. Future work will have to elucidate the exact nature of the cathodic corrosion process, in terms of the exact chemical nature of the corrosion intermediate (presumably a cation-stabilized surface hydride) and how it relates to the high anisotropy of the corrosion process.

## Experimental Methods

### Materials and Chemicals

EC-AFM experiments were carried out in a home-build electrochemical AFM cell (as shown in Figure S1 in the Supporting Information) made of polychlorotrifluoroethylene (PCTFE). All cell components and the electrolyte reservoir were cleaned in freshly prepared piranha (3:1 v/v H_2_SO_4_ (96%, Merck Suprapur) and H_2_O_2_ (35%, Merck Suprapur) for over 2 h, followed by at least five times rinsing and boiling with ultrapure water (Milli-Q, 18.2 ΜΩ cm).

Electrolytes were made from ultrapure water, high-purity reagents HClO_4_ (60%), H_2_SO_4_ (96%), and NaClO_4_ (99.99%) from Merck Suprapur. Before each experiment, the electrolytes were first purged with argon (Air Products, 5.7) for at least 30 min to remove air from the solution. Afterward, argon flow was carefully introduced to the atmosphere above the electrolyte.

Disk-type polycrystalline Pt and Pt(111) single-crystal electrodes (2 mm diameter) were used as working electrodes (MaTecK), respectively. The polycrystalline Pt electrode was annealed with a butane flame and quenched with Milli-Q water before assembling into the electrochemical AFM cell (Figure S1). The Pt(111) single-crystal electrode was prepared by repeated cycles of mild etching (large-amplitude sinusoidal voltammetry, LASV) from 2 V to −2 V for 124 cycles at 50 Hz in electrolyte (2.5 M CaCl_2_ plus concentrated HCl) and rinsed thoroughly with ultrapure water, and flame annealed several times according to the Clavilier method.^40^ This procedure has been shown to deliver a clean surface of Pt(111) single-crystal electrode with a minimum amount of contamination.^10^ After corrosion, the roughened Pt working electrode was annealed by inductive heating in an inert all-quartz tube filled with a stream of hydrogen, which is an efficient way to convert a mildly corroded Pt electrode surface back to an etching pattern free surface. A coiled platinum wire was used as counter electrode, and a reversible hydrogen electrode (RHE, Mini HydroFlex, Gaskatel) was employed as the reference electrode. The Autolab PGSTAT204 potentiostat and a Booster (10 A) were coupled with the AFM (JPK NanoWizard 4) to control the electrochemical conditions during the experiments. The current density shown here represents the measured current normalized to the geometric area of the working electrode.

### In Situ Electrochemical Atomic Force Microscopy (EC-AFM) Measurements

AFM scan rate was 1 Hz, and all the images were obtained using tapping mode, to minimize the damage to the electrode and AFM probe. The tips used were purchased from Bruker (SNL, resonance frequency: 65 kHz; spring constant: 0.35 N/m). The “hydrogen region” and “(bi)sulfate region” are extremely sensitive to the crystallographic structure of the Pt electrode since the adsorption energies for both species depend on the geometry of the particular adsorption sites, and hence, the CV in sulfuric acid is highly sensitive to (changes in) the surface structure. Therefore, CV characterization of the Pt surface after corrosion was performed in sulfuric acid solution. Prior to cathodic corrosion of Pt electrodes, the surface quality and cleanliness were checked by cyclic voltammograms and AFM images in 0.1 M H_2_SO_4_. The limits of the potential sweep were imposed between 0.05 and 0.65 V_RHE_ and 0.05–0.85 V_RHE_ for polycrystalline Pt and Pt(111) single-crystal electrode, respectively, to prevent any possible change caused by anodic corrosion. Next, the Pt electrodes were subjected to constant cathodic potentials during various time periods in acid solution, without and with the addition of cations (NaClO_4_) in HClO_4_ for comparative studies. Subsequently, the cyclic voltammograms of Pt electrodes were recorded in 0.1 M H_2_SO_4_ and the surface morphologies were imaged by AFM for comparison. All AFM images were collected at a potential of 0.5 V_RHE_ in the Pt double-layer region to avoid any possible change to the surface and damage to the AFM probe.

## Data Availability

The data sets generated during and/or analyzed during the current study are available from the corresponding author on reasonable request.

## Supporting Information Available

Schematics of the EC-AFM setup; further EC-AFM images of polycrystalline Pt and Pt(111) (PDF)
