# Supplementary material for: In Situ EC-AFM Study of the Initial Stages of Cathodic Corrosion of Pt(111) and Polycrystalline Pt in Acid Solution
Source: J Phys Chem Lett. 2023 May 24;14(21):4997–5003. doi: 10.1021/acs.jpclett.3c00579 (PMC10240529; doi:10.1021/acs.jpclett.3c00579)
Supplement: Supplementary file 1 — jz3c00579_si_001.pdf [file jz3c00579_si_001.pdf]

Supporting Information

In situ EC-AFM study of the initial stages of cathodic corrosion of Pt(111) and polycrystalline Pt in acid solution

Xiaoting Chen[‡+], Marc T.M. Koper[+*]

[‡]School of Materials Science and Engineering, Beijing Institute of Technology, Beijing 100081, P. R. China

[+]Leiden Institute of Chemistry, Leiden University, PO Box 9502, 2300 RA, Leiden, the Netherlands; email:m.koper@chem.leidenuniv.nl

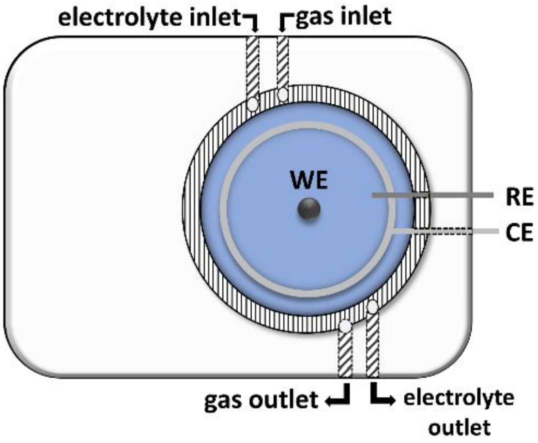

Figure S1. A schematic draw of the top-view of the home-build *in situ* electrochemical atomic force microscopy (EC-AFM) cell.

Figure S1 depicts the home-built *in situ* EC-AFM cell which was designed and improved based on our previous reports[1]. The whole cell was made from a polychlorotrifluoroethylene (PCTFE) cube including the designed components of electrolyte and gas inlet/outlet, respectively. The cell utilizes a three-electrode configuration. The counter electrode (CE), reference electrode (RE) and working electrode (WE) could be mounted to the set up after cleaning and/or annealing procedure. The feeding electrolyte is pre-purged with argon for at least 30 minutes to remove air from the solution before flowing into the EC-AFM cell through Teflon tube. The individual Ar flow introduced by gas inlet and outlet at room temperature helps to keep an oxygen free atmosphere above the electrolyte facilitating the electrochemical signal collection before and after cathodic corrosion.

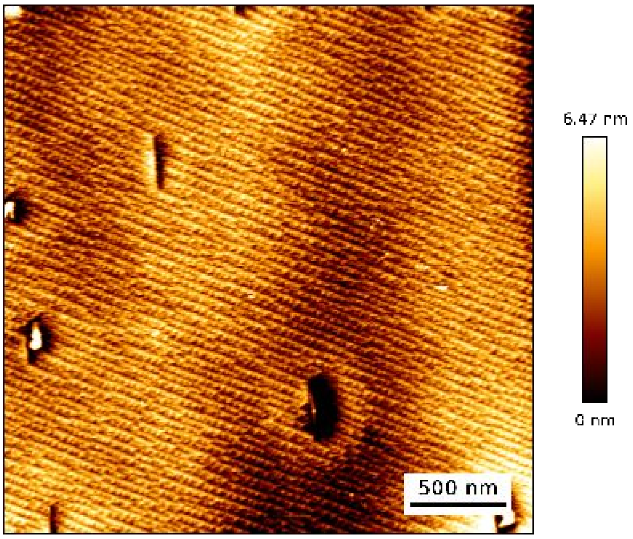

Figure S2. AFM height images of a polycrystalline Pt electrode electrode surface obtained in the *in situ* EC-AFM cell.

Figure S2 shows the original topography of a polycrystalline Pt electrode imaged in a large frame of ca. 3×3 μm, showing what appear to be defect structures with a large lateral and height size most likely caused by flame annealing.

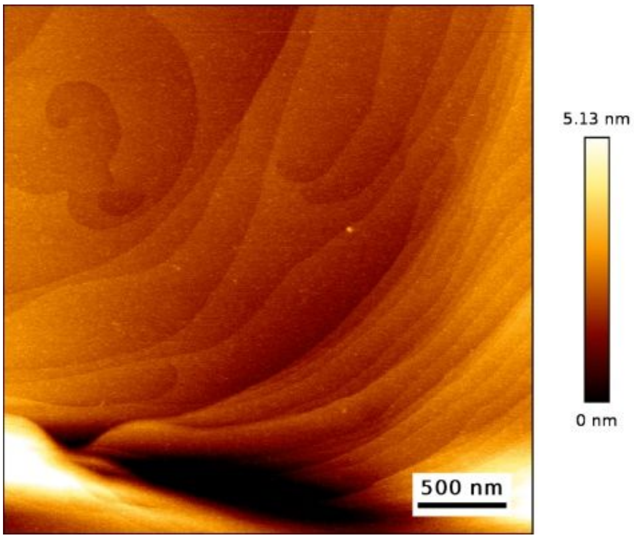

Figure S3. AFM height image of a pristine Pt(111) electrode surface obtained in the *in situ* EC-AFM set up.

Figure S3 shows the original topography of a Pt(111) single crystal electrode imaged in a large frame of ca. 3×3 μm, showing flat terraces with various widths (10~500 nm) separated by steps/defects and is comparable to the state-of-the-art reported Pt(111) surfaces reported by *in situ* EC-STM[2,3] and EC-AFM[4], respectively, in acid electrolytes.

References

(1) Deng, X.; Galli, F.; Koper, M. T. In situ electrochemical AFM imaging of a Pt electrode in sulfuric acid under potential cycling conditions. *J. Am. Chem. Soc.* **2018**, 140, 13285-13291 .

(2) Sashikata, K.; Furuya, N.; Itaya, K. In situ electrochemical scanning tunneling microscopy of single - crystal surfaces of Pt (111), Rh (111), and Pd (111) in aqueous sulfuric acid solution. *J. Vac. Sci. Technol. B* **1991**, 9, 457-464.
(3) Jacobse, L.; Huang, Y.-F.; Koper, M. T.; Rost, M. J. Correlation of surface site formation to nanoisland growth in the electrochemical roughening of Pt (111). *Nat. Mater.* **2018**, 17, 277-282.
(4) Lopes, P. P.; Li, D.; Lv, H.; Wang, C.; Tripkovic, D.; Zhu, Y.; Schimmenti, R.; Daimon, H.; Kang, Y.; Snyder, J. Eliminating dissolution of platinum-based electrocatalysts at the atomic scale. *Nat. Mater.* **2020**, 19, 1207-1214.
